# Ethnobotanical, phytochemical and antimicrobial activity of *Halexylon salicornicum* (Ramth) as a graze and promising shrub against selected animal microbes

**DOI:** 10.1016/j.sjbs.2022.103328

**Published:** 2022-05-29

**Authors:** Rehab M.A. El-Desoukey, Fawziah M. Albarakaty, Nurah M. Alzamel, Mashail N. AlZain

**Affiliations:** a Microbiology and Immunology Department, National Research Centre, Giza, Egypt; b Department of Biology, Faculty of Applied Science, Umm Al-Qura University, Makkah, Al Mukarramah, P.O. Box: 715, Saudi Arabia; c Biology Department, Faculty of Science and Humanities in Al Quwai’iyah, Shaqraa University, Shaqraa 11961, Saudi Arabia; d Department of Biology, College of Sciences, Princess Nourah bint Abdulrahman University, Riyadh 11451, Saudi Arabia

**Keywords:** Natural antimicrobial, Halexylon salicornicum, Ramth, Grazing herbs, Shrub, Animal pathogen, Camel, Ethnobatanical

## Abstract

Folk medicine, including plants, has been utilized for humans and animals since the dawn of civilization. Because of the widespread problem of antimicrobial resistance around the world, one of the mainly significant challenges in microbiological research is to achieve a replacement antibiotic with the fewest adverse effects. Camel and ruminant grazing were provided by the wild shrub *Halexylon salicornicum* (Ramth). However, despite prior research demonstrating its antimicrobial action against human diseases, no investigations on its antimicrobial activity against animal pathogens have been conducted to far. The goal of this study is to investigate whether *Halexylon salicornicum* aqueous and solvent extracts have antimicrobial activity on a variety of animal pathogens isolated from cattle and poultry using the agar well diffusion method (*Enterococcus faecium, Shigella flexneri, Bacillus Cereus, Klebsiella pneumoniae, Staphylococcus aureus, Escherichia coli, Streptococcus pyogens, Pseudomonas aerogenes, Salmonella typhimurium, and Candida albicans*) moreover it's ethnobotanical and phytochemical. All of the extracts tested had antimicrobial efficacy against tested strains and included bioactive chemicals, particularly the acetone extract, had the highest antibacterial activity. As a result, it can be stated that *Halexylon salicornicum* is a promising important shrub that might be used as a natural antimicrobial alternative for animals or as a food additive.

## 1 Introduction

Half of the deaths in animals by infectious diseases are due to microbes especially that lead to diarrhea the leading cause of illness and mortality in poor nations. Most of these cases due to (*Salmonella and E.coli)* (Alavijeh et al., 2012, ElDesoukey, 2015, El-Desoukey, 2017). In neonates are commonly associated with (*C. parvum, E. coli,* R*ota and* C*oronavirus)* which can lead to outbreaks (Merck Sharp and Dohme Corp, 2014). In addition to *Candida albicans* is the main cause of systemic fungal infections (Sofos et al., 1998).In dairy cows the most common cause of clinical mastitis is mainly *Staphylococci* and *Klebsiella* pneumonia (Thorberg et al., 2009, Schuh and Weinstock, 1985). *Bacillus cereus* is the main cause of necrotizing placentitis (Munoz et al., 2006). Due to the high cost of chemical antibiotics, long degradation period, it's side effects moreover the increase of antimicrobial resistance, especially in animal products, therefore a lot of researchers trying to find an alternative natural antimicrobial from plants and herbs (Abd El-Latif et al., 2002, Buchanan et al., 2008, Duraipandiyan et al., 2006). The chemical composition of the plant plays an important role as an alternative medicinal therapy, especially in it's bioactive compound such as alkaloids, flavonoids tannins and saponins (Kanife and Odesanmi, 2012, Tariq et al., 2018). Also, a lot of people in developing countries believe in and use the herbs in folk medicine for both man and animals which do not require prolonged or expense treatment (Aziz et al., 2018).

*Haloxylon salicornicum* is a shrub or undershrub belonging to the Chenopodiaceae family that is present in many countries in Asia and Africa including the Kingdom of Saudi Arabia (El-Wahab et al., 2014, Phondani et al., 2015). *Haloxylon salicornicum* can be physiologically and ecologically adapt and tolerate the water limitation, salty land and high temperature, each part of the shrub is traditionally used for animal food (fruiting top, seeds), human food during drought especially in India and shrub wood as fuel (Ajabnoor et al., 1984). *H. salicornicum* has been approved to have anti-inflammatory, anti-diabetic and Antiseptic activities (Awaad et al., 2014). These all activities in addition to hepatoprotective are supposed to be due to the shrub's secondary metabolites as triterpenoids, alkaloids, tannins, glycosides, flavonoids and tannins (Eman, 2011, Karimi et al., 2011). The organic extracts of the shrub showed significant antibacterial against some bacteria of human origin (*Salmonella typhi, Staphylococcus aureus, Micrococcus luteus, Sarcina ventriculi and Bacillus subtilis*) in addition to antifungal effect against some fungi of human origin (*Penicillium chrysogenum, Aspergillus flavus, Candida albicans, Aspergillus fumigates and Candida tropicalis*) (Dash et al., 2008, Kim and Van Ta, 2011).These antimicrobial activities due to its phytochemical content (tannins, alkaloids, saponins and flavonoids) have a lot of useful pharmacological effects (Hammer et al., 2001, Shankar and Kumar, 1984). However *H. salicornicum* is not used globally (Ajabnoor et al., 1984). It has been used as important fodder for camels, sheep and goats in some countries including Kingdom of Saudi Arabia (Singh et al., 2015). Also it has been used in folk medicine for some diseases of man and animals (Arshad et al., 2002).

To far, however, there have been no studies that have investigated the antimicrobial activity of *H. salicornicum* extract against pathogens from animal sources especially as a grazing herb that is consumed by camels and small ruminants select it for eating by instinct that my resume the use due to the Zoopharmacognosy as animals medicate themselves by selecting natural substances (plants, herbs, clay and insects) to reduce the risky effect of pathogens (Kapadia et al., 2014, Attardo and Sartori, 2003). Especially as the safety for therapeutic uses of aqueous extract of the shrub *H. salicornicum* has been demonstrated and can be used without any significant toxicity (Ullah et al., 2019). So this research aimed to inspect the phytochemical, ethnobotanical and antimicrobial effect of *H. salicornicum* against pathogens of animal origin to give more attention to this neglected shrub.

## 2 Materials and methods

### 2.1 Materials and reagents

**2.1.1.** Reagents used for extraction (Ether, Ethanol and Acetone) and reagents used for phytochemical tests (Ferric Chloride, Glacial Acetic Acid, Alcoholic Potassium Hydroxide, Sulfuric Acid, chloroform, ammonia and Formaldehyde) were purchased from Arkan Group, Fisher chemical® in addition to media and antimicrobial used for antimicrobial assay (Nutrient Agar, Potato Dextrose Agar, Mueller Hinton Agar, Peptone Water, Mc Farland BSS 0.5, Ciprofloxacin, tetracycline, cefpodoxime, Erythromycin, Gentamycin, Augmentin and Nystatin) were also purchased from Arkan Group, scharlab®.

#### 2.1.1 Collection of shrub

*H. salicornicum* aerial parts were collected during the flowering season in March 2018 from Valley of the Quwai', in Al Quwai’iyah, a large Province beside Riyadh Province, Saudi Arabia.

#### 2.1.2 Bacterial and fungal strains

The bacterial and fungal strains used in this study (*Enterococcus faecium, Shigella flexneri, Bacillus Cereus, Klebsiella pneumoniae, Staphylococcus aureus, Escherichia coli, Streptococcus pyogens, Pseudomonas aerogenes, Salmonella typhimurium, and Candida albicans*) were isolated from some clinical cases in poultry and cattle farms from Cairo Province, Egypt, outskirts belonging to first author previous research. Where those samples have been identified biochemically in Microbiology and Immunology Department/ National Research centre / Egypt.

### 2.2 Ethnobotanical

2.2.1. The shrub description and taxonomy have been done by Dr/ Nurah. Alzamel, Assistant Professor of plant ecology, Biology Department, Faculty of Science and humanities in Al Quwai’iyah, Shaqraa University, where the shrub is kept in the herbarium of the same department, Shaqraa University, with voucher number (BSQS#48) the description was compared to (Migahid, 1978, Chaudhary, 1999).

2.2.2. Traditional and therapeutic uses of shrubs were gathered from previous studies, textbooks, websites, journals, symposia, periodicals, and databases that manage medicinal plants used to treat human diseases in Saudi Arabia, the Arabian Peninsula, and other areas of the world. Where the accuracy of English/Arabic and Arabic/English dictionaries has been confirmed (Aati et al., 2019).

### 2.3 Extraction methods

#### 2.3.1 Aqueous extraction

The aerial parts of the shrub *H.salicornicum* were washed three times with clean distilled water and air-dried in the shade before being ground into a powder in a blender. Cold aqueous extracts were prepared using the infusion method (Thakur et al., 2011, Nayak et al., 2011), while hot aqueous extracts were prepared using the decoction method (Thakur et al., 2011). (Shetty et al., 2008). The extracts were then stored at 4 °C until they were required.

#### 2.3.2 Solvent extraction

The grounded shrub *H. salicornicum* was extracted using (ether, ethanol and acetone) by solvent extraction method according to (Patil et al., 2009). Then the extracts were reserved at 4 °C till to be used.

### 2.4 Phytochemical analysis:

#### 2.4.1 Tannins Test:

A modified technique was employed to validate the presence of tannins in the extracts, according to (Kanife, 2012). A few drops of Ferric chloride reagent were combined with 3 ml of extract. The presence of tannins was detected by the appearance of a blue-black coloration.

#### 2.4.2 Alkaloids Test:

To the 5 ml of extract, a few drops of Marqus reagent (made from 0.5 ml of Formaldehyde and 5 ml of concentrated H2SO4) were added. The presence of alkaloids was detected by turbidity (Ajoku et al., 2015).

#### 2.4.3 Saponins Test:

For 5 min, vigorously shake the combination of 3 ml of *H. salicornicum* extracts and 10 ml of distilled water in a test tube. Then leave the test tube for 30 min to see the creation of honeycomb foam, which indicates the presence of saponins (Cho et al., 2003, Clarke, 1975).

#### 2.4.4 Flavonoids Test:

The flavonoids test was carried out following (Doss et al., 2011). A yellow tint formed when 2 ml of the extract was mixed with Alcoholic KOH (0.5 mol.), indicating the presence of flavonoids.

#### 2.4.5 Glygosides Test:

1 ml of conc. H2SO4 was added to 0.5 g of ground *H. salicornicum*, which had been mixed in 2 ml glacial acetic acid with one drop of ferric chloride solution. The presence of Glycosides was indicated by a brown ring (Antia et al., 2010).

#### 2.4.6 Anthraquinones test

A mixture of 1 g of the *H. salicornicum* powder and 20 ml of chloroform was heated for 5 min in a steam bath. Then the mixture while hot was filtered and let to be cool. Added equal volume of 10% ammonia solution to the filtrate. Shaking the mixture the appearance of bright pink color in the upper layer of the mixture indicated the presence of Anthraquinones (Cho et al., 2003, Clarke, 1975).

#### 2.4.7 Sterols test

A quantity of 1 ml of H2SO4 was added to the extract. The appearance of a brownish-red ring in the contact line between the two liquids shows the presence of sterols (Bankole et al., 2016, Senhaji et al., 2005).

### 2.5 Antimicrobial screening

The investigation of the antimicrobial activity *H. salicornicum* (Ramth) aqueous and solvent extracts was done by agar well diffusion method against bacterial and fungal strains isolated from cattle and poultry farms (*Enterococcus faecium, Shigella flexneri, Bacillus Cereus, Klebsiella pneumoniae, Staphylococcus aureus, Escherichia coli, Streptococcus pyogens, Pseudomonas aerogenes, Salmonella typhimurium, and Candida albicans*). Inoculated with the studied strains on petri dishes containing 20 ml of nutrient agar (for bacteria) and malt extract (for fungi) and standardized using McFarland number 0.5 to yield 1–2 X 107 cfu/ml standard inoculums. Wells of about 5 mm in diameter were drilled into the media, which were then filled with 50 μl of aqueous and solvent extracts at a concentration of 5 mg/ml. All plates were incubated for 1–3 days at 37 °C. The antibacterial efficacy was confirmed by measuring the inhibitory zone in millimeters. Distilled water (100 μl) was employed as a negative control for antibacterial and antifungal screening, whereas the control was found to be positive for both antibacterial and antifungal screening as part of an antimicrobial sensitivity test. (NCCLS, 1993). MS Excel was used to obtain the mean and the standard deviation for each sample.

### 2.6 Determination of minimum inhibitory concentration (MIC)

The *H. salicornicum*-susceptible bacterial and fungal strains were inoculated into sterile peptone water and incubated overnight at 37 °C. After that, 9 ml of sterile peptone water was added to sterile test tubes, followed by 1 ml of the various concentrations of extracts, and finally 0.3 ml of the culture of the studied strains. Only peptone water and the extract were used in the control. For Bacteria and Fungi, the inoculated and control tubes were incubated at 37 °C and 25 °C for 24 h and 48 h, respectively, before being checked for turbidity. The MIC was determined to be the lowest concentration that produced no turbidity (Adetitun et al., 2013).

### 2.7 Determination of the minimum bactericidal or fungicidal concentration (MBC/MFC)

After incubation, samples from the MIC test tubes that did not display turbidity were streaked out with sterile cotton swabs on solidified Nutrient Agar (Bacteria) and Potato Dextrose Agar (Fungi) plates and incubated at 37 °C and 25 °C, respectively. After 24 and 48 h of incubation, MBC was determined as the lowest concentration of the extract that showed no growth on plates, suggesting bactericidal or fungicidal action (Adetitun et al., 2013).

### 2.8 Antimicrobial susceptibility test

The disc diffusion method was used for the analysis. Antibiotic discs were utilized (Ciprofloxacin, tetracycline, cefpodoxime, Erythromycin, Gentamycin, Augmentin and Nystatin). The Mueller Hinton agar plates were seeded with the tested strains before adding the standard antibiotic discs with sterile forceps and incubating at 37 °C and 25 °C for 24 h, respectively. The antibiotics' inhibition zones were measured to the closest millimeters (mm) and categorized as susceptible (+) or resistant (−) (Anibijuwon and Udeze, 2009).

## 3 Results

### 3.1 Ethnobotanical studies

#### 3.1.1 The studying area

**Fig. 1:**
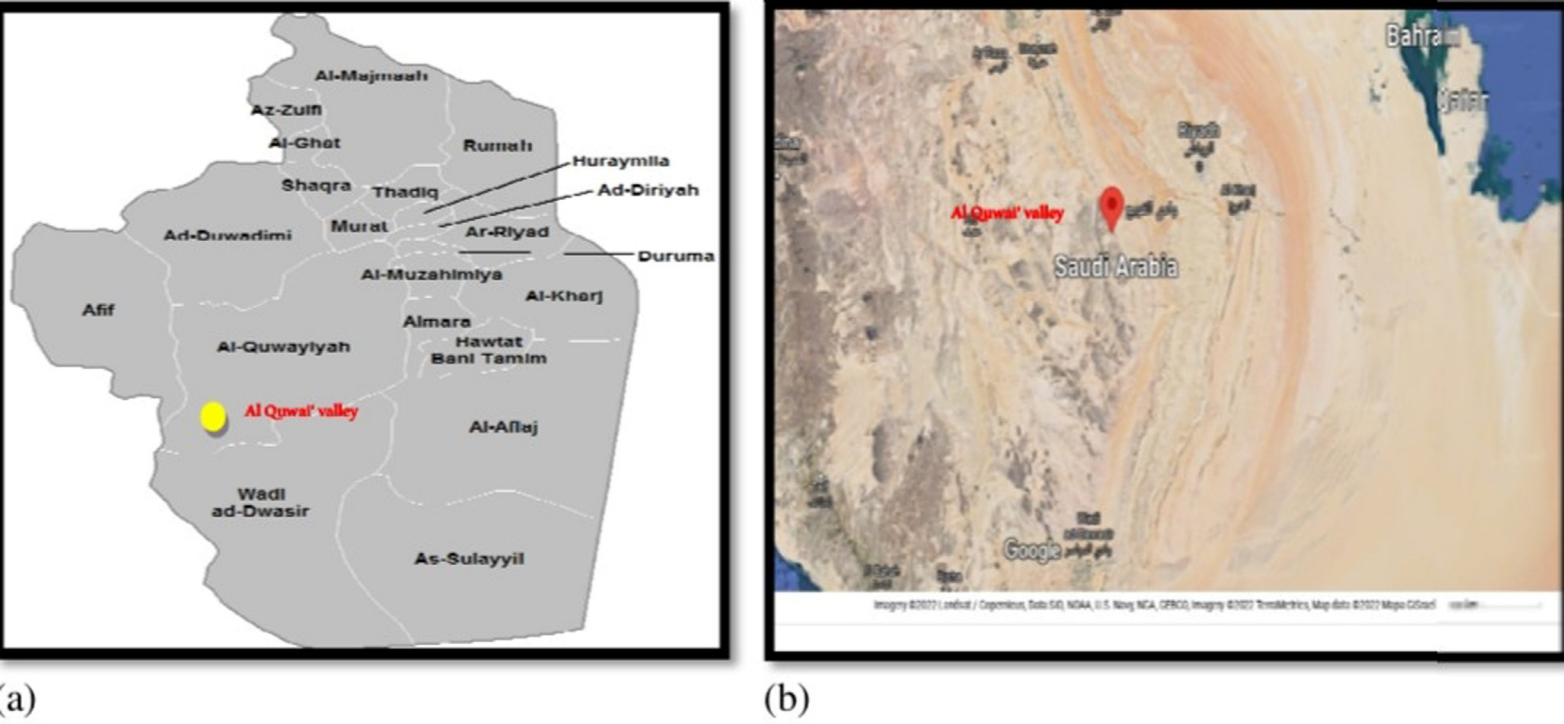
(a and b). This is a figure demonstrated the location of Al-Quway'iyah Province and Al Quwai' valley in Saudi Arabia map.

The area of the present study is Al Quwai' valley in Al-Quway'iyah or Al Gwei'iyyah (Arabic name: القويعية) is a large Province beside Riyadh Province, Saudi Arabia. Where it is located 165 km by road southwest of Riyadh. It had a population of 126,161 people according to the 2010 census.The Province of Al-Quway'iyah is considered one of the largest provinces of the kingdom of Saudi Arabia that is located on a flat plain surrounded by mountain ranges from three sides the north and west or south, or to the west is the famous supply chain of fiery configuration, and the eastern desert is flat and wide sedimentary form, which is the desert of Hadba and the desert Jala so it represents the tip of the Arabian Shield rock. It occupies the point of convergence between the ancient pyramids and the calcareous limestone rocks, and the most famous valley in Al-Quway'iyah is Al Quwai' valley (Wadi Al Quwai',) which descends from the west to the east and then rush to the Hadba desert and spread through it and the name of Al-Quway'iyah derived from the name of the valley. The valley contains a lot of wild herbs and shrubs used for animal grazing, fuel and folk medicine (Fig. 1 a and b).

#### 3.1.2 Plant description, folk uses and medicinal uses

Table 1 demonstrated that *Haloxylon salicornicum* (Moq) Bunge ex Boiss is a shrub of family Chenopodiaceae. Considered a promising shrub used for both man and animals in folk medicine in several countries and also used for grazing, a lot of studies confirmed its importance as antidiabetic, antioxidant, antinflammatory, anticancer and antimicrobial for some human pathogens.

**Table 1:** This is a table demonstrate ethnobotanical of *Haloxylon salicornicum*.

Ethnobotanical items		References
Family name	*Chenopodiaceae*	(Shankar and Kumar, 1984, Al-Sodany et al., 2013, Shawky and Alzamel, 2020)
Species name	*Haloxylon salicornicum*	(Shankar and Kumar, 1984, Al-Sodany et al., 2013, Shawky and Alzamel, 2020)
Common name	Ramth in Arabic رمث	(Al-Sodany et al., 2013, Shawky and Alzamel, 2020)
Plant life form	Shrublets	(Shankar and Kumar, 1984, Al-Sodany et al., 2013, Shawky and Alzamel, 2020)
Plant specimen voucher number	BSQS#48	Herbarium of Biology Department, Faculty of Science and humanities in Al Quwai’iyah, Shaqraa University
Favrouble soil for plant	Salty land, mound basis, salty depressions.	(Shawky and Alzamel, 2020, El-Shaer, 2010)
Traditional uses for man	Valuable source of food especially seeds in drought condition in arid region Used the plant ash for cloth washing. Used the wood for fuel	(El-Shaer, 2010, Ali and Qaiser, 2001, Al-Ani and Jawad, 1974, Singh et al., 2003).
Traditional uses for animals	Used for camel and small ruminants grazing with very high concentrated food	(Shankar and Kumar, 1984, Ibnoaf, 1987, Singh et al., 2009, Ali et al., 2009, Chaudhary et al., 2003).
Folk Medicinal uses for man	Used for internal ulcer healing and insect stings. Used for dysmenorrheal by drinking as tee Used the smog for cold in Saudi Arabia. Used for hepato-biliary treatment Used for sexual disorders	(Singh et al., 2003, Chopra et al., 1956, Anonymous., 1959, Ahmad and Eram, 2011, Stewart, 1972).
Folk Medicinal uses for animals	Used in some region as ethno-veterinary medicine as anti-parasitic for lice, myiasis and tick treatment Fly repellent	(Farooq et al., 2008, Raza, 2013).
Studies on plant	Plant extracts have anti diabetic effect. Antifungal effect on some human pathogenic fungi Plant derivative has lipoxygenase inhibition effect anticoagulant effect of plant extract plant derivative has anti-tuberculosis activities plant extract has hepatoprotective effects Free radicals isolated from plant extract have inhibition activity. Free radicals from plant extract have antioxidants and anti-inflammatory effect Plant extract has inhibition of uterine contraction Plant extract has antibacterial effect on some human pathogens. Plant extract has antiparasitic effect on some animal parasites. Increase body gain in camel as food additives. The plant extract has anticancer effect. The plant has antipyretic, antioxidant and anti-inflammatory effect.	(Ajabnoor et al., 1984, Shabana et al., 1990, Ferheen et al., 2005a, Awaad et al., 2001, Ferheen et al., 2005b, Alqasoumi et al., 2012, Bibi et al., 2010, Ibrahim et al., 2011, Saleem et al., 2013, Abdallah and El-Ghazali, 2013, Saini et al., 2009, Ullah et al., 2019, Aala et al., 2021).

### 3.2 Phytochemical analysis

**Fig 2:**
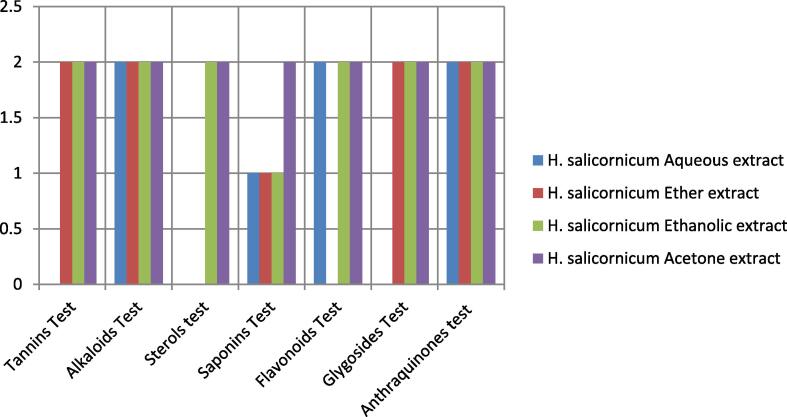
This is a figure demonstrate the phytochemical profile of *H. salicornicum* different extracts. *0 = absence of phytochemical compound, 1 = traces of phytochemical and 2 = presence of phytochemical compound.

As shown in Table 2 and Fig. 2, all extracts contained phytochemical compounds, particularly acetone and ethanol extracts, which contained all bioactive compounds (Alkaloids, Saponins, Tannins, Glycosides, Sterols, Anthraquinones, and Flavonoids), whereas Glycosides, Tannins, and Flavonoids were absent in the aqueous extract. The ether extract is devoid of flavonoids and sterols. According to the information in Table 2 and Fig. 2, the optimum solvents for extracting phytochemical compounds from the shrub *H. salicornicum* are acetone and ethanol.

**Table 2:** This is a table demonstrate the phytochemical constituents of *H. salicornicum*.

Phytochemical tests	Plant extracts			
	*H. salicornicum* Aqueous Extract	*H. salicornicum* Ether Extract	*H. salicornicum* Ethanolic Extract	*H. salicornicum* Acetone Extract
Tannins Test	_	+	+	+
Alkaloids Test	+	+	+	+
Sterols test	_	_	+	+
Saponins Test	±	±	±	+
Flavonoids Test	+	_	+	+
Glygosides Test	_	+	+	+
Anthraquinones test	+	+	+	+

*(+) = presence of phytochemical compound, (_) = absence of phytochemical compound), (±) = traces.

### 3.3 The antimicrobial assay, MIC, MBC and

Table 3, Table 4, Table 5, Table 6, Table 7 show that all types of shrub *H. salicornicum* extracts have significant antimicrobial activity against the examined animal pathogenic strains when compared to the standard antimicrobial. As shown in Table 3, Table 7, acetone extract has the highest significant antimicrobial activity, followed by ethanol extract, ether, and aqueous extracts. These findings, combined with those in Table and Fig. 2, revealed that acetone and ethanol are the optimum solvents for producing *H. salicornicum* extracts with potent antibacterial, antifungal properties.

**Table 3:** This is a table demonstrate the antimicrobial activity of *Haloxylon salicornicum* (Ramth)aqueous and organic extracts against some animal pathogen in (mm).

Type of microorganism		Types of extract					Mean	ST.DEV.	Control negative
		Aqueous extract		Organic extracts					Distelled water
		Hot aqueous extract	Cold aqueous extract	Ether	Acetone	Ethanol			
Gram negative	*Ps.aerogens*	0	0	0	16	24	8	11.31	0
	*E coli*	0	0	17	36	0	10.6	15.99	0
	*Enterococcus*	0	0	19	39	25	16.6	16.80	0
	*Shigella*	27	0	0	18	26	14.2	13.42	0
	*Salmonella*	0	0	16	38	23	15.4	16.14	0
Gram positive	*K. pneumonia*	17	13	0	23	29	16.4	10.99	0
	*Bacillus*	0	0	0	17	19	7.2	9.88	0
	*S.pyogens*	0	0	0	19	21	8	10.97	0
	*S. aureus*	0	0	0	18	26	8.8	12.37	0
fungi	*C albicans*	0	0	0	23	26	9.8	13.46	0

*Mean = the average for each sample, ST.DEV. = the standerded deviation for each sample.

**Table 4:** This is a table demonstrate the MIC and MBC/MFC of the ether extract of the *Haloxylon salicornicum*.

Examined strains	Concentrations of ether extract (mg/mL)							
	5	25	50	75	100	150	MIC	MBC/MFC
*E coli*	++	+	+	_	_	_	75 mg/ml	100 mg/ml
*Salmonella*	++	+	+	_	_	_	75 mg/ml	100 mg/ml
*Enterococcus*	+	_	_	_	_	_	25 mg/ml	50 mg/ml
*Ps.aerogens*	++	++	+	+	+	+	_	_
*Shigella*	+	+	+	+	+	+	_	_
*Bacillus*	++	++	+	+	+	+	_	_
*S. aureus*	++	++	+	+	+	+	_	_
*S.pyogens*	+	+	+	+	+	+	_	_
*K. pneumonia*	+	+	+	+	+	+	_	_
*Candida albicans*	++	++	++	+	+	+	_	_

*(+) = turbid (microbial growth), (++) = very turbid (high microbial growth), (_) = no turbidity (no microbial grwoth).

**Table 5:** This is a table demonstrate the MIC and MBC/MFC of the ethanol extract of the *Haloxylon salicornicum*.

Examined strains	Concentrations of ethanol extract (mg/mL)							
	5	25	50	75	100	150	MIC	MBC/MFC
*E coli*	++	+	+	+	+	+	_	_
*Salmonella*	++	+	_	_	_	_	50 mg/ml	75 mg/ml
*Enterococcus*	++	+	_	_	_	_	50 mg/ml	75 mg/ml
*Ps.aerogens*	++	+	_	_	_	_	50 mg/ml	75 mg/ml
*Shigella*	++	+	_	_	_	_	50 mg/ml	75 mg/ml
*Bacillus*	++	++	+	_	_	_	75 mg/ml	100 mg/ml
*S. aureus*	++	+	_	_	_	_	50 mg/ml	75 mg/ml
*S.pyogens*	+	+	_	_	_	_	50 mg/ml	75 mg/ml
*K. pneumonia*	+	_	_	_	_	_	25 mg/ml	50 mg/ml
*Candida albicans*	+	+	_	_	_	_	50 mg/ml	75 mg/ml

*(+) = turbid (microbial growth), (++) = very turbid (high microbial growth), (_) = no turbidity (no microbial grwoth).

**Table 6:** This is a table demonstrate the MIC and MBC/MFC of the acetone extract of the *Haloxylon salicornicum*.

Examined strains	Concentrations of acetone extract (mg/mL)							
	5	25	50	75	100	150	MIC	MBC/MFC
*E coli*	_	_	_	_	_	_	5 mg/ml	25 mg/ml
*Salmonella*	_	_	_	_	_	_	5 mg/ml	25 mg/ml
*Enterococcus*	_	_	_	_	_	_	5 mg/ml	25 mg/ml
*Ps.aerogens*	++	++	+	_	_	_	75 mg/ml	100 mg/ml
*Shigella*	+	+	+	_	_	_	75 mg/ml	100 mg/ml
*Bacillus*	++	++	+	_	_	_	75 mg/ml	100 mg/ml
*S. aureus*	++	++	+	_	_	_	75 mg/ml	100 mg/ml
*S.pyogens*	+	+	+	_	_	_	75 mg/ml	100 mg/ml
*K. pneumonia*	++	+	_	_	_	_	50 mg/ml	75 mg/ml
*Candida albicans*	++	+	_	_	_	_	50 mg/ml	75 mg/ml

*(+) = turbid (microbial growth), (++) = very turbid (high microbial growth), (_) = no turbidity (no microbial grwoth).

**Table 7:** This is a table demonstrate antimicrobial Activity of Standard Gram negative and gram positive antibiotics on the examined strains.

Control positive antimicrobial agents	Examined strains									
	*Escherichia* *Coli*	*Salmonella* *Typhimurium*	*Enterococcus*	*Ps.aerogens*	*Shigella*	*Bacillus*	*S. aureus*	*S.pyogens*	*K. pneumonia*	*Candida albicans*
Ciprofloxacin (5 µg)	20 (S)	30 (S)	35 (S)	31 (S)	- (R)	34 (S)	25 (S)	- (R)	38 (S)	Nt
Cefpodoxime (10 µg)	19 (S)	16 (S)	- (R)	- (R)	- (R)	20 (S)	27 (S)	15 (S)	22 (S)	Nt
Tetracycline (30 µg)	31 (S)	29 (S)	25 (S)	19 (S)	- (R)	17 (S)	20 (S)	- (R)	27 (S)	Nt
Erythromycin (ERY) (5 µg)	- (R)	- (R)	- (R)	- (R)	- (R)	22 (S)	32 (S)	- (R)	23 (S)	Nt
Gentamycin (10 µg)	- (R)	- (R)	- (R)	- (R)	- (R)	25 (S)	31 (S)	- (R)	29 (S)	Nt
Augmentin (30 µg)	- (R)	- (R)	- (R)	- (R)	- (R)	14 (S)	35	19 (S)	27 (S)	Nt
Nystatin (30 µg)	Nu	Nu	Nu	Nu	Nu	Nu	Nu	Nu	Nu	16 (S)

*(S) = Susiptable, (R) = Resistent and (Nu) = Not used.

## 4 Discussion

The Kingdom of Saudi Arabia is a large, waterless area with an extent of approximately 2 km that covers a substantial portion of the Arabian Peninsula and is characterized by diverse ecosystems and plant species (Abdel Khalik et al., 2013). Saudi Arabia's geomorphology, like that of the Arabian Peninsula, is a primordial massif in which geologic formation developed in lockstep with the mountains. The climate in Saudi Arabia varies greatly from the coast to the interior. Along the shore, higher moisture levels are associated with milder temperatures, whereas the interior is marked by dehumidification and high temperatures (Country Profile: Saudi Arabia, 2006). Even though Saudi Arabia lacks permanent gutters or lakes, “Wadis” may be found all around the nation. In this article, the term “Wadi” refers to a non-endless stream whose runoff is dependent on rainfall. It happens frequently, although Wadis have been underused for decades. Wadis are one of the most noticeable desert areas in arid regions, with physiographic irregularities and associated variances in plant species allocation (Kassas and Girgis, 1964). Wadi al-Quwai'a“ is one of the most significant Wadis in Saudi Arabia's Al-Quway'iyah Province's western section. The valley is rich in wild plants and bushes that are utilized for grazing, firewood, and traditional medicine (Fig. 1 a and b).

Saudi Arabia's foliage is one of the most diverse biodiversities in the Arabian Peninsula, with a sizable inheritable coffer of crops and medicinal plants (Rahman et al., 2004). The distribution of living forms is closely linked to geomorphology and landscape (Kassas and Girgis, 1965, Zohary, 1973, Orshan, 1986, Fakhireh et al., 2012). The structure of life forms in Wadis reflects typical desert vegetation, with therophytes and chamaephytes accounting for the bulk of species. The foliage of Wadis in general isn't consistent; it changes from season to season, depending on the humidity level (Siddiqui and Al-Harbi, 1995). Numerous factors, such as geographical position, physiographic characteristics, and environmental effects, influence the development, maturity, renewal, and allocation of plant populations in the Wadis (Shaltout and El-Sheikh, 2003, Kürschner and Neef, 2011). Thousands of traditional plants are quietly discovered uncultivated or farmed in a variety of countries, according to ethnobotanical inspections. These plants are often ignored or neglected across the world, although they've been shown to have major uses on a local or worldwide level (Alatar et al., 2012). Furthermore, the majority of these plants are suitably adapted to a variety of frontier growth constraints, such as drought or salty environments, contribute to nutrition, and play an important role in naturalistic curative therapies (Korkmaz and Ozcelik, 2013). *Haloxylon salicornicum (Moq)* Bunge ex Boiss is a Chenopodiaceae family shrub. The species has evolved to survive in parched locations where water is scarce and nutrients are few (Ajabnoor et al., 1984). This species is used as a meal, food aid, medicine, and in the regeneration of waterless regions that have become degraded. As has been demonstrated in Table 1.

However, no studies have examined the antimicrobial activity of *H. salicornicum* extract against microbes from animal sources, especially because it is a grazing herb that camels and small ruminants eat by instinct, and it may be used again due to Zoopharmacognosy, which states that animals medicate themselves by selecting natural substances (plants, herbs, clay, and insects) to reduce the risky effects of pathogens (Kapadia et al., 2014, Attardo and Sartori, 2003). Especially since the aqueous extract of the shrub *H. salicornicum* is safe for medicinal purposes and may be administered without causing substantial harm (Ullah et al., 2019). As a result, the goal of this study was to examine the ethnobotanical, phytochemical, and antibacterial effects of *H. salicornicum* against pathogens of animal origin to give this underutilized shrub more attention.

As shown in Table and Fig. 2, all extracts contained phytochemical compounds, particularly acetone and ethanol extracts, which contained all bioactive compounds (Alkaloids, Saponins, Tannins, Glycosides, Sterols, Anthraquinones, and Flavonoids), whereas Glycosides, Tannins, and Flavonoids were absent in the aqueous extract. The ether extract is devoid of flavonoids and sterols. According to the information in Table and Fig. 2, the optimum solvents for extracting phytochemical compounds from the shrub *H. salicornicum* are acetone and ethanol. Much prior research has demonstrated the therapeutic value of bioactive chemicals derived from plants (Ahmad et al., 2008, Cowan, 1999, Darout et al., 2000, Eloff, 1998, Emad et al., 2009, Karaman et al., 2003, Malu et al., 2009, Mothana and Lindequist, 2005, Lin et al., 1999, Parekh and Chanda, 2008).

Additionally, Table 3, Table 4, Table 5, Table 6, Table 7 show that all types of shrub *H. salicornicum* extracts have significant antimicrobial activity against the examined animal pathogenic strains when compared to the standard antimicrobial. As shown in Table 3, Table 7, acetone extract has the highest significant antimicrobial activity, followed by ethanol extract, ether, and aqueous extracts. These findings, combined with those in Table and Fig. 2, revealed that acetone and ethanol are the optimum solvents for producing *H. salicornicum* extracts with potent antibacterial and antifungal properties. Much prior research (Awaad et al., 2014, Shabana et al., 1990, Ferheen et al., 2005b, Bibi et al., 2010, Abdallah and El-Ghazali, 2013, Aala et al., 2021) However, the best of our knowledge, no studies have been conducted to date that has investigated the antimicrobial effect of *H. salicornicum* extracts on animal pathogens, except for what has been reported for the first time in this article, so our results in addition to the previous data that have been mentioned by (Mathur et al., 2011, Saini et al., 2009) about this promising shrub that has great value as concentrates in rations of camel and small ruminants, moreover enhancing milk production, which led to the suggestion of utilizing the *H. salicornicum* shrub as a food supplement in poultry and cow rations.

## 5 Conclusion

So it could be concluded that the *H. salicornicum* shrub considered an important promising grazing shrub that was present naturally in arid rejoins can tolerate the salty land and inadequate water containing high value of nutrition to animals requires more attention in both veterinary and agricultural aspects and recommended to be introduced as a natural source of animal food as growth promoters and natural prophylactic antimicrobial, especially in poultry and cattle farms instead of chemical antimicrobial that were added to the animal rations in farms with no side effects or residues in animal products that also will help to decrease the bacterial resistance for human by consuming the safe animal products. Also, the results confirmed and recommended the use of acetone and ethanol as the best solvents for extraction of bioactive compounds from *H. salicornicum* shrub.

## Declaration of Competing Interest

The authors declare that they have no known competing financial interests or personal relationships that could have appeared to influence the work reported in this paper.
